# Multifunctional Hydrogel Microneedles (HMNs) in Drug Delivery and Diagnostics

**DOI:** 10.3390/gels11030206

**Published:** 2025-03-15

**Authors:** Hossein Omidian, Sumana Dey Chowdhury

**Affiliations:** Barry and Judy Silverman College of Pharmacy, Nova Southeastern University, Fort Lauderdale, FL 33328, USA

**Keywords:** hydrogel microneedles (HMNs), transdermal drug delivery, biosensing diagnostics, smart and responsive materials, clinical translation

## Abstract

Hydrogel microneedles (HMNs) have emerged as a transformative platform for minimally invasive drug delivery and biosensing, offering enhanced bioavailability, controlled drug release, and real-time biomarker detection. By leveraging swelling hydrogels, nanomaterial integration, and stimuli-responsive properties, HMNs provide precision medicine capabilities across diverse therapeutic and diagnostic applications. However, challenges remain in mechanical stability, as hydrogel-based MNs must balance flexibility with sufficient strength for skin penetration. Drug retention and controlled release require optimization to prevent premature diffusion and ensure sustained therapeutic effects. Additionally, biosensing accuracy is influenced by variability in interstitial fluid extraction and signal transduction. Clinical translation is hindered by regulatory hurdles, scalability concerns, and the need for extensive safety validation in human trials. This review critically examines the key materials, fabrication techniques, functional properties, and testing frameworks of HMNs while addressing these limitations. Furthermore, we explore future research directions in smart wearable MNs, AI-assisted biosensing, and hybrid drug–device platforms to optimize transdermal medicine. Overcoming these barriers will drive the clinical adoption of HMNs, paving the way for next-generation patient-centered therapeutics and diagnostics.

## 1. Introduction

The field of transdermal drug delivery and biosensing has witnessed significant progresse with the development of hydrogel microneedles (HMNs), a minimally invasive technology that enables controlled drug release, interstitial fluid (ISF) extraction, and real-time biomarker detection. Unlike traditional hypodermic needles or passive transdermal patches, HMNs leverage hydrogel-forming polymers to penetrate the skin, absorb ISF, and facilitate sustained drug diffusion or biosensing interactions [1,2,3]. These systems provide several advantages over conventional delivery and diagnostic platforms, including enhanced patient compliance, reduced systemic toxicity, and improved drug bioavailability, making them highly suitable for chronic disease management, vaccination, wound healing, and cancer therapy [1,4,5,6,7,8].

The concept of microneedles dates back to the early 20th century, with Dr. Ernst Kromayer pioneering microneedle-like techniques in dermatology for scar treatment and pigmentation disorders. The first mention of microneedle use appeared in 1921, while interest in transdermal drug delivery through microneedles began to grow in the 1960s. However, it was not until the late 1990s that microfabrication advancements enabled the development of silicon microneedles for enhanced drug delivery [9]. Since then, microneedle technology has rapidly evolved, incorporating diverse materials, fabrication techniques, and biomedical applications, paving the way for modern HMNs.

The primary mechanism of HMNs involves polymer-based microneedle arrays that swell upon contact with skin moisture, forming a conduit for therapeutic agents or biomarker analysis [5,10,11,12]. This technology allows for tunable mechanical properties, precise drug-loading capabilities, and multi-responsive release kinetics. Moreover, the integration of smart and stimuli-responsive hydrogels has enabled on-demand drug administration, responding to physiological cues such as pH, glucose fluctuations, or enzymatic activity [4,13,14]. In diagnostics, HMNs have emerged as a powerful alternative to invasive blood sampling techniques, offering continuous glucose monitoring, electrochemical biosensing, and smartphone-integrated biomarker detection [15,16,17].

The rapid expansion of microneedle-based technologies has led to a surge in academic interest, with research increasingly focusing on their potential in transdermal drug administration, vaccines, and biomolecule delivery for skin-related conditions [18]. While their benefits are well-documented, challenges remain regarding scalability, mechanical stability, and drug-loading capacities. Addressing these limitations early on is crucial for optimizing the clinical translation of microneedle platforms.

HMNs are fabricated using a variety of techniques, including micromolding, photopolymerization, 3D printing, and enzyme-mediated crosslinking, each tailored to optimize mechanical integrity, drug retention, and biosensing efficiency [19,20,21,22]. Recent advancements have further expanded the scope of microneedle applications, incorporating various types such as solid, coated, hollow, dissolvable, hydrogel, swellable, and porous microneedles, each with unique properties depending on materials and fabrication methods [23]. The versatility of microneedles extends beyond drug delivery to include diagnostics, tissue engineering, cancer research, and wound care, making them a promising tool in modern biomedical applications.

Advanced material engineering has also introduced nanoparticle-enhanced HMNs, integrating gold nanoparticles, graphene oxide, and metal–organic frameworks (MOFs) to improve drug solubility, bioavailability, and precision targeting [15,24,25]. Additionally, wearable and wireless MN patches are being developed to facilitate real-time health monitoring, with potential applications in chronic disease management and point-of-care diagnostics [17,26]. Emerging precision medicine strategies, such as the acupoint–target-organ–ganglion approach, have further refined drug targeting, improving therapeutic efficacy through specific drug concentration at intended sites [27]. Innovations in biocompatible materials and mechanical properties are expected to enhance microneedle performance while ensuring patient safety and comfort.

Despite their advantages, the widespread clinical translation of HMNs remains hindered by challenges in drug release precision, mechanical stability, scalability, biosensing accuracy, and regulatory approval [28,29,30,31,32,33]. Issues such as burst release, unintended drug leakage, and inconsistent swelling behavior affect performance, while manufacturing complexities and high production costs limit commercialization. Additionally, regulatory frameworks for combination drug–device MN systems remain undefined, slowing clinical adoption and necessitating large-scale multi-center trials to validate efficacy and safety.

This review presents a comprehensive analysis of the key materials, fabrication strategies, biosensing capabilities, and drug delivery mechanisms of HMNs. It further explores their limitations, regulatory considerations, and emerging trends, emphasizing the potential of next-generation hybrid, AI-driven, and personalized MN platforms to transform transdermal therapeutics and diagnostics.

## 2. Key Materials for HMNs

HMNs’ success in transdermal drug delivery and diagnostics depends largely on the composition of the hydrophilic polymers, crosslinkers, nanoparticles, and functional bio-additives that form their structure. These materials determine the mechanical properties, drug-loading efficiency, biodegradability, and responsiveness to stimuli.

### 2.1. Crosslinked and Hydrophilic Polymers: The Backbone of HMNs

HMNs rely on hydrophilic polymers for their mechanical strength, swelling properties, and controlled drug release. These polymers ensure that the microneedles can penetrate the skin, absorb interstitial fluid, and deliver therapeutic agents effectively.

#### 2.1.1. Synthetic Polymers for Structural Integrity and Drug Delivery

Synthetic polymers provide stability, tunability, and high drug-loading capacity in HMNs. Poly(ethylene glycol) (PEG), available in molecular weights of 10,000 Da, enhances hydrophilicity and flexibility, ensuring optimal drug diffusion through the microneedle matrix [1,34]. Similarly, poly(methyl vinyl ether-co-maleic acid) (PMVE/MA) with sodium bicarbonate significantly improves swelling behavior and drug retention, making it ideal for sustained-release formulations [1,10].

Poly(acrylic acid-co-maleic Acid) (PAMA) and polyvinyl alcohol (PVA) (1:4 ratio, crosslinked at 90 °C for 30 min) create a stable matrix that swell upon contact with skin moisture, allowing for rapid drug release [35]. Additionally, methacrylated hyaluronic acid (MeHA) is widely used for biofilm penetration, aptamer-based biosensing, and targeted drug delivery due to its excellent biocompatibility and modification flexibility [36].

#### 2.1.2. Natural and Hybrid Polymers for Biocompatibility

Natural polymers enhance the biocompatibility and biodegradability of HMNs. Silk fibroin methacrylate provides a strong structural network while enabling the delivery of bioactive molecules such as alpha-MSH, a treatment for vitiligo [37]. Additionally, methacryloyl chitosan (CSMA) hydrogels, on the other hand, are widely used for psoriasis treatment, allowing for the controlled release of methotrexate and nicotinamide [38].

### 2.2. Functionalized Nanoparticles and Crosslinking Agents for Enhanced Performance

HMNs are often reinforced with crosslinkers and nanoparticles to improve mechanical strength, drug-loading efficiency, and response to stimuli.

#### 2.2.1. Crosslinking Agents for Structural Reinforcement

Chemical crosslinking enhances HMNs’ mechanical properties, ensuring they can penetrate the skin without breaking. Na_2_CO_3_ (3% *w*/*w*) plays a crucial role in regulating pH and modifying crosslinking density to optimize swelling and stability [1]. Dopamine-functionalized hydrogels contribute to biosensing capabilities by facilitating redox-based diagnostics [26].

For high-dose drug delivery, Gantrez S-97, PEG (10,000 Da), and Na_2_CO_3_ are combined to produce super-swelling HMNs, which provide the rapid absorption of interstitial fluid and controlled release of therapeutic agents [39]. Phenylboronic acid-based hydrogels introduce an additional level of control by enabling glucose-responsive insulin delivery through reversible phenylborate ester crosslinking, making them highly effective for diabetes treatment [13,40]. An aqueous blend of poly(vinyl alcohol) (PVA), polyvinylpyrrolidone (PVP), crosslinked with citric acid, was able to deliver methotrexate, albendazole, and sildenafil citrate to treat different disease conditions [41,42,43].

#### 2.2.2. Functionalized Nanoparticles for Targeted Therapy

Nanoparticles improve HMNs’ ability to carry and release drugs in a controlled manner. Similarly, tetrakis(1-methyl-4-pyridinio)porphyrin (TMPyP)-loaded PLGA nanoparticles inside enzyme-mediated hyaluronic acid-tyramine (HAT) hydrogels enable photodynamic therapy in melanoma treatment by targeting cancer cells with light-activated drug release [4]. For antibiotic-resistant infections, mono-(6-diethylenetriamine-6-deoxy)-beta-cyclodextrin (mbeta-CD) is incorporated into HMNs to deliver celastrol, which has potent antimicrobial properties [12]. Dopamine-conjugated hyaluronic acid hydrogels integrated with PEDOT:PSS and Ag-Pt nanoparticles further enable real-time glucose monitoring and pH biosensing, improving diabetes management and metabolic disorder tracking [15,30].

### 2.3. Smart and Responsive Polymers for Controlled Drug Release and Biosensing

Smart polymers in HMNs introduce responsiveness to physiological conditions, allowing for precise drug release based on pH, temperature, or external stimuli.

#### 2.3.1. Temperature- and pH-Responsive Polymers

Poly(N-Isopropylacrylamide) (pNIPAM) undergoes phase transitions at body temperature, enabling controlled insulin release for diabetic patients without the need for continuous injections [44,45]. For inflammation treatment, taurine-loaded Prussian blue nanoparticles in methacrylate-based hyaluronic acid HMNs are designed to release anti-inflammatory agents upon exposure to acidic and photothermal stimuli, making them useful for chronic wound healing [46].

#### 2.3.2. Electrically and Light-Responsive Hydrogels

Electrically conductive and light-sensitive HMNs provide additional control over drug delivery. HMNs containing black phosphorus (BP) microspheres and pNIPAM introduce near-infrared (NIR)-triggered drug release for precise insulin administration in diabetic patients [44]. Moreover, HMNs composed of polydopamine@polypyrrole enable light-responsive photothermal effects by absorbing near-infrared light and converting it into heat for killing bacteria [47].

### 2.4. Hybrid Hydrogels for Sustained Drug Release

A hybrid form of HMN provides a controlled and prolonged drug release profile while minimizing toxicity and adverse effects.

#### 2.4.1. Crosslinked Hybrid Polymers

Poly(lactide-co-glycolide) (PLGA) tips combined with poly(vinyl alcohol) (PVA) and polyvinylpyrrolidone) (PVP) hydrogel bases create hybrid microneedles for sustained amphotericin B release, improving antifungal treatments [48]. Poly(methylvinylether-co-maleic acid) crosslinked with pectin enhances bioadhesion and controlled drug diffusion, making it useful for transdermal drug administration [11].

#### 2.4.2. Drug Reservoirs and Inclusion Complexes

To improve drug solubility, beta-cyclodextrin drug reservoirs are integrated into HMNs for delivering telmisartan for treating hypertension and curcumin for anticancer therapy [49,50]. HMNs embedded in collagen type-I cryogels are optimized for ocular drug delivery, providing antibacterial treatment for eye infections [51]. Additionally, HMNs also used several other reservoir systems such as PEG reservoir, lyophilized reservoir, and compressed tablet reservoir [2,49,52].

Table 1 presents key materials and additives used in HMNs, highlighting their notable properties. Synthetic polymers such as PVA, PVP, PEG, and Gantrez S-97 dominate, offering mechanical strength, swelling ability, and biocompatibility. Natural polymers like chitosan, alginate, and hyaluronic acid (HA) emphasize biodegradability and bioadhesion. Crosslinked materials (e.g., MeHA, GelMA, DexMA) provide enhanced gel stability and tunable mechanical properties. Stimuli-responsive polymers, such as pNIPAAm and Carbopol, enable temperature- and pH-sensitive drug release. Nanomaterials (graphene oxide, silver nanoparticles) contribute conductivity and antimicrobial properties. Overall, the table highlights a balance between biocompatibility, mechanical strength, and smart drug release properties, suggesting a trend toward hybrid, responsive, and multifunctional HMN formulations.

## 3. Fabrication Techniques for HMNs

Various fabrication techniques have been developed to optimize drug loading, tunable release kinetics, and improved biocompatibility. These methods range from polymerization and crosslinking strategies to 3D printing, micromolding, and smart nanomaterial integration. This section explores the diverse manufacturing approaches used in HMN fabrication, highlighting their advantages and applications in drug delivery and diagnostics.

### 3.1. Polymerization and Crosslinking Techniques

One of the most straightforward fabrication techniques for HMNs involves polymerization, where hydrogel formation and drug loading occur simultaneously. For example, polymer blending methods, such as combining PMVE/MA and PEG in a 1:3 ratio, optimize drug encapsulation for acyclovir delivery [1]. More advanced polymerization techniques involve multi-step crosslinking to fine-tune drug release properties. Vat photopolymerization is a popular method used to fabricate GelMA microneedles, where controlled exposure times dictate needle height and mechanical strength [19]. Similarly, microwave-assisted crosslinking has been employed to create 1,4-butanediol diglycidyl ether (BDDE)-crosslinked microneedles, reducing fabrication time while improving swelling kinetics and drug retention [20].

Crosslinking plays a vital role in enhancing the stability and mechanical strength of HMNs. Silk fibroin methacrylate polymerization has been employed to ensure uniform drug loading and slow-release kinetics for peptide-based therapeutics [37]. Another strategy involves esterification-based crosslinking, used in poly(methyl vinyl ether-co-maleic acid) and pectin to form microneedles, which enhances bioadhesive properties and controlled drug diffusion [11].

For glucose-responsive microneedles, phenyl-borate ester crosslinking has been employed, allowing self-regulated insulin release in response to fluctuating glucose levels [13]. Similarly, tartaric acid crosslinking in polyvinyl-based microneedles improves the mechanical properties and solubility of sildenafil citrate and telmisartan-loaded HMNs [49,52].

### 3.2. 3D Printing and Micromolding Techniques

#### 3.2.1. Micromolding for Standardized Microneedle Arrays

Micromolding is one of the most widely used techniques for mass-producing HMNs with uniform size and structure. For example, micromolding has been applied to fabricate HMNs for alpha-arbutin skin applications, ensuring high reproducibility and enhanced dermal absorption [35]. Another example is dextran-methacrylate biosensor microneedles, where micromolding provides stable crosslinking for long-term glucose monitoring [21].

**Figure 1:**
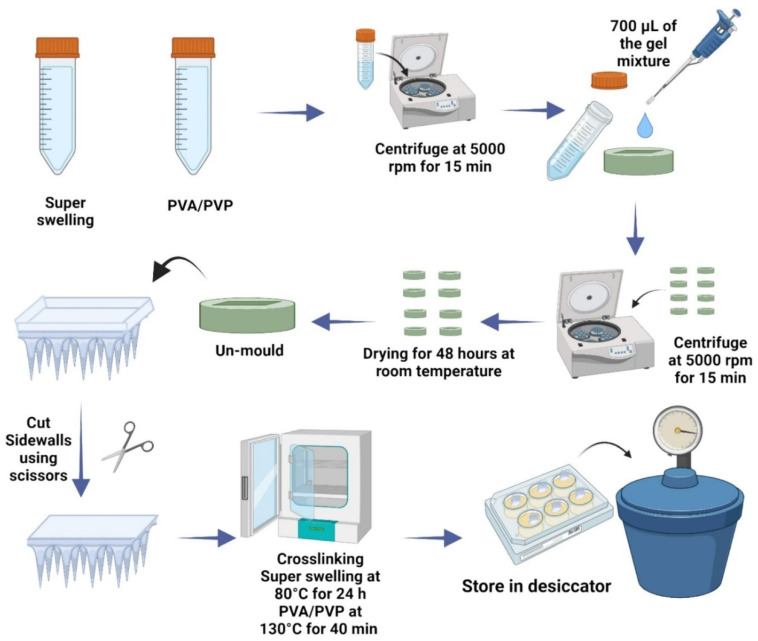
Schematic workflow of HFMAP fabrication using micromolding method and single-step crosslinking [57].

Micromolding is also employed in the production of hydrogel-forming microneedle patches (HFMAPs) for controlled transdermal risperidone delivery, ensuring efficient skin penetration and prolonged drug release [57]. This technique allows for cost-effective and scalable production, making it ideal for commercial applications. Figure 1 presents the schematic workflow for the preparation of HFMAPs using the micromolding method.

#### 3.2.2. 3D Printing for High-Resolution Microneedle Fabrication

Three-dimensional printing has transformed microneedle fabrication by allowing precise control over geometry, drug distribution, and mechanical properties. Digital light processing (DLP) 3D printing is another advanced technique that enables high-resolution GelMA microneedle fabrication, optimizing drug loading for antibiotics such as amoxicillin [65].

Additionally, high-precision digital light processing (H-P DLP) 3D printing systems are used to fabricate HMNs with sharp protrusions and microporous structures. These HMNs can perform multifunctional tasks, including drug delivery and detection, with minimal invasiveness [89]. The flexibility of 3D printing also enables the creation of triple-responsive HMNs that are non-cytotoxic, exhibiting sensitivity to pH, temperature, and glucose levels. This allows for more precise on-demand drug delivery [90].

### 3.3. Smart and Enzyme-Mediated Microneedle Fabrication

#### 3.3.1. Enzyme-Crosslinked Hydrogels for Tunable Properties

Enzyme-mediated microneedle fabrication provides customizable mechanical strength and drug release kinetics. Enzyme-crosslinked hyaluronic acid-tyramine (HAT) hydrogels have been designed for TMPyP photodynamic therapy, allowing for precise control over stiffness and degradation rates [4]. Similarly, hydrolyzed poly(methylvinylether/maleic anhydride) crosslinked with PEG enhances microbial resistance and mechanical stability, making it ideal for long-term transdermal applications [91].

Another innovative approach involves triblock amphiphiles with enzyme-cleavable hydrophobic end groups, enabling sustained drug release [6]. For smart biosensing applications, dopamine-functionalized hydrogels allow for redox-based ketone sensing, integrating machine learning-assisted monitoring for diabetes management [26].

#### 3.3.2. Functional Nanomaterial Integration for Enhanced Performance

The integration of functional nanomaterials into HMNs has improved their mechanical, electrochemical, and biosensing capabilities. Black phosphorus and pNIPAM microspheres, fabricated using capillary microfluidics, have been integrated into pyramid-shaped microneedles, allowing NIR-triggered insulin release for diabetic therapy [44]. Similarly, MIL-100(Fe) nanoparticles embedded in a PVA-chitosan matrix enable sequential metformin release, optimizing diabetes treatment [54].

For biosensing applications, graphene oxide-blended microneedles crosslinked via gamma radiation provide high electrical conductivity, allowing for smart drug release based on electrical stimulation [58]. Additionally, Au/Cu_2_O nanoparticles integrated into methacrylated hyaluronic acid (MeHA) HMNs have been used for enzyme-free electrochemical glucose sensing, improving real-time diabetes monitoring [16].

### 3.4. Hybrid and Specialized Fabrication Techniques

**Figure 2:**
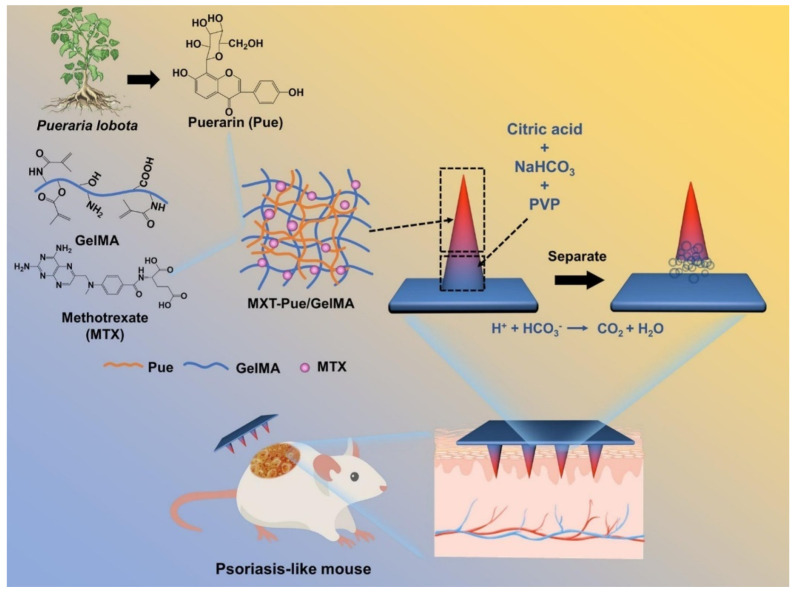
Schematic illustration of MTX-loaded Pue/GelMA HMNs for psoriasis treatment. Adopted with permission [71].

Effervescent microneedles provide enhanced penetration and rapid drug diffusion, especially for topical and systemic treatments. Effervescent HMNs loaded with methotrexate and puerarin generate CO_2_ gas upon insertion, separating the microneedle tips and ensuring sustained psoriasis treatment over 10 days (Figure 2) [71]. Similarly, the freeze-thaw fabrication of polydopamine@polypyrrole microneedles enhances mechanical stability and water absorption, optimizing bacterial biofilm degradation [47].

Table 2 presents various microneedle fabrication methods, detailing their advantages, limitations, cost, and biocompatibility. Some methods, like micromolding and freeze-thaw cycling, are cost-effective and highly biocompatible, making them suitable for large-scale production. Others, such as 3D printing and photopolymerization, offer precision and customization but require expensive equipment and specialized materials. Enzyme-mediated crosslinking and chemical crosslinking allow for tunable mechanical properties but may involve high costs or the need for careful purification. Innovative approaches, such as conductive hydrogel integration and hybrid microneedles, enhance functionality but introduce fabrication complexity. Meanwhile, osmosis-powered MNs and effervescent mechanisms improve ISF extraction and drug delivery, providing simple scalable alternatives. Each method balances fabrication efficiency, cost, and biocompatibility based on its intended biomedical application.

## 4. Functional Properties of HMNs

The performance of HMNs in drug delivery and diagnostics depends on various physical and chemical properties, including drug permeation, release kinetics, mechanical robustness, swelling behavior, biosensing sensitivity, and biocompatibility.

### 4.1. Drug Permeation and Release Kinetics

#### 4.1.1. Enhanced Skin Permeation and Transdermal Drug Absorption

HMNs significantly improve drug permeability by increasing skin penetration efficiency and diffusion rates. For example, SmartFilm HMNs incorporating rifampicin achieved a four-fold increase in skin deposition (~80 ± 7 µg) and high permeation efficacy (~500 ± 22 µg) [94]. Similarly, acyclovir-loaded HMNs resulted in a 39-fold increase in transdermal drug permeation, delivering 75.56 ± 4.2% of the drug within 24 h [1].

The effectiveness of drug cocktails was also demonstrated in tuberculosis therapy, where HMNs loaded with rifampicin, ethambutol, and pyrazinamide showed variable permeation rates, with rifampicin achieving 75% (3.64 mg) and ethambutol reaching 47% (46.99 mg) transdermal penetration [2]. Similarly, caffeine-loaded HMNs improved transdermal absorption by 6.1-fold, offering a promising delivery system for pediatric applications [95].

#### 4.1.2. Sustained and Controlled Drug Release

Sustained drug release is a crucial property of HMNs, as it allows for prolonged therapeutic effects and reduces the need for frequent administration. Dexamethasone-loaded microneedles provided 90% drug release over six days and showed first-order drug release, ensuring long-term anti-inflammatory effects [6]. Likewise, methotrexate and nicotinamide microneedles achieved an 80% release in 24 h, effectively reducing psoriasis severity in mice [38].

Similarly, celastrol-loaded HMNs enabled sustained drug diffusion, leading to reduced bacterial infections and inflammatory responses in infected tissues [12]. Phenylboronic acid-crosslinked HMNs offered glucose-sensitive swelling, ensuring self-regulated insulin delivery for diabetes management [13].

For oral drug alternatives, telmisartan-loaded HMNs achieved 83.3 ± 2.4% transdermal permeation, improving bioavailability by 20-fold compared to oral formulations [49]. Meanwhile, sildenafil citrate microneedles delivered 80% of the drug transdermally, enhancing patient compliance and reducing side effects [52].

### 4.2. Mechanical Strength and Swelling Behavior

#### 4.2.1. Structural Integrity and Skin Penetration Efficiency

Mechanical stability is essential for microneedles to maintain structural integrity during insertion and to facilitate efficient drug delivery. Gelatin methacryloyl (GelMA) microneedles showed no breakage at 0.3 mm displacement, while 1,4-butanediol diglycidyl ether (BDDE)-crosslinked microneedles improved structural durability and swelling capacity [19,20].

Moreover, PEG-PMVE/MA microneedles demonstrated outstanding insertion properties, with drug release controlled by swelling that preserved mechanical stability [1]. A highly resilient super-swelling microneedle array, capable of withstanding an insertion force of up to 324.9 N/array (equivalent to 361 needles per array), efficiently penetrated the skin, swelled significantly, and remained intact during removal. This process optimized transdermal absorption while ensuring sustained drug diffusion [39].

Glycerol-crosslinked microneedles for photodynamic therapy showed a 14% height reduction under a 20N compression force, confirming structural robustness [14]. Furthermore, pectin-based HMNs exhibited only a 22.33% height loss under axial compression, confirming robust mechanical performance [11].

#### 4.2.2. Hydrogel Swelling and Drug Diffusion

HMNs swell upon skin penetration, increasing the surface area for drug absorption and controlled diffusion. Poly(acrylic acid-co-maleic acid) (PAMA) and polyvinyl alcohol (PVA)microneedles fully swelled within four hours, ensuring enhanced drug retention and controlled permeation [35]. Similarly, PVA-based microneedles for albendazole delivery achieved over 400% swelling capacity, maintaining skin integrity for 24 h post-application [53]. Sildenafil citrate microneedles exhibited a swelling capacity of 300–700%, enabling improved drug permeation with pain-free administration [52].

### 4.3. Biosensing Sensitivity and Selectivity

#### 4.3.1. Glucose and Lactate Monitoring for Diabetes Management

**Figure 3:**
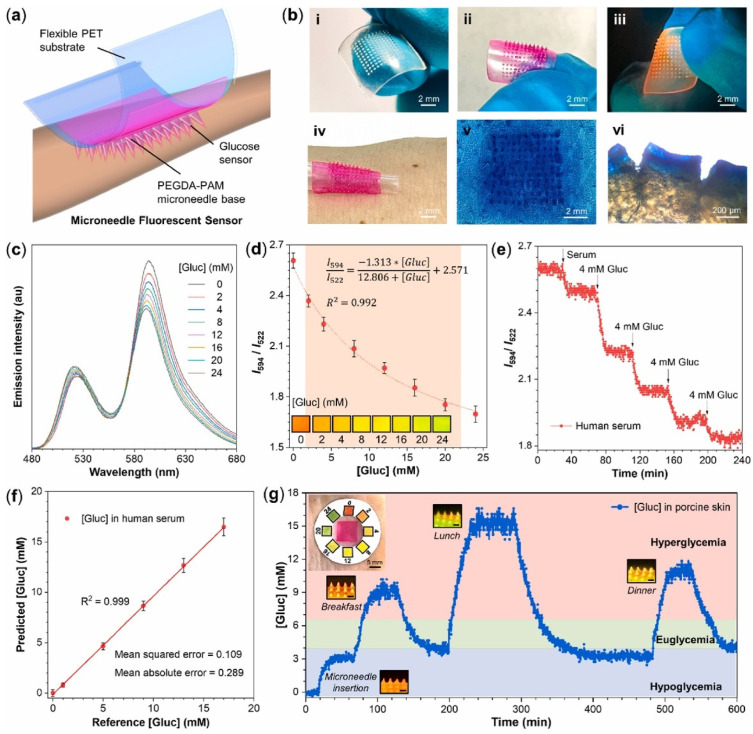
(**a**) Scheme of the wearable microneedle fluorescent sensor patch. (**b**) Images of (**i**) microneedle base; (**ii**) microneedle sensor; (**iii**) fluorescent microneedle sensor excited by UV light; (**iv**) microneedle sensor patch on human skin; (**v**) holes in pork skin after the injection of microneedle sensor; (**vi**) side-view holes observed by microscope. (**c**) Emission spectra of microneedle sensors with various [Gluc] (0–24 mM) in artificial ISF, recorded by fiber optic devices. λex = 450 nm. (**d**) The FRET ratio (I595/I520) of microneedle sensors as a function of [Gluc]. Inset: photos taken under a 450 nm blue light. The error bars represent the standard deviations of three different measurements. (**e**) The response of FRET ratio to [Gluc] changes in human serum over 240 min. (**f**) The correlation between predicated and reference [Gluc] in human serum. Each data point and standard deviations were obtained from 60 reading points. (**g**) Continuous glucose monitoring of microneedle sensor patch in ex vivo porcine skin over 600 min. The red, green, and blue regions of [Gluc] correspond to hyperglycemia, euglycemia, and hypoglycemia, respectively. Insets: Photographs of the microneedle sensor patch on porcine skin and its fluorescent response to [Gluc] changes under a 450 nm blue light. Scale bar: 500 μm [78].

HMNs are used for biosensing applications, allowing for real-time biomarker detection with high accuracy. Glucose and lactate sensor microneedles detected glucose levels between 1 and 8 mM and lactate concentrations of 0.1–12 mM, enabling effective chronic disease monitoring [5]. For glucose detection, dextran-methacrylate biosensor microneedles maintained stable readings for up to 10 days, ensuring long-term reliability [21]. Similarly, FRET-based HMNs demonstrated a sensitivity of 0.029 mM^−^^1^, with a detection limit of 0.193 mM and a response time of 7.7 min [78]. Figure 3 represents the schematic diagram of a wearable microneedle sensor patch for continuous glucose monitoring.

#### 4.3.2. Non-Invasive Biomarker and Disease Monitoring

Microneedles have also been applied for real-time biomarker detection in diseases such as cancer, neurodegenerative disorders, and infectious diseases. HMNs functionalized with glypican-1 antibodies successfully captured tumor-derived exosomes, enabling early colorectal cancer detection [96].

For wound healing applications, HMNs loaded with dopamine-conjugated polymers provided 93% accuracy in pH detection, monitoring skin acidity changes in real time [30]. Meanwhile, interstitial fluid sampling microneedles demonstrated 52.4% efficiency for isoniazid detection and 54.4% for theophylline, proving effective for therapeutic drug monitoring [62].

### 4.4. Biocompatibility, Targeted Therapy, and Safety

#### 4.4.1. Minimal Invasiveness and High Patient Compliance

HMNs are biocompatible, non-toxic, and minimally invasive, making them suitable for long-term therapeutic applications. The biocompatible polyvinylpyrrolidone (PVP)-based microneedle patch is used for smart drug delivery through electrical stimulation [58]. HMNs for amoxicillin delivery demonstrated high mechanical strength (0.1 N/needle) and effectively inhibited bacterial growth, confirming their antimicrobial efficacy [65]. Additionally, beta-carotene-loaded microneedles for dermatological applications increased drug release seven-fold, with no signs of skin irritation or toxicity [97]. HMNs for chronic wound management with doxycycline effectively reduced biofilms and enhanced angiogenesis, accelerating wound healing [98].

#### 4.4.2. Tissue Regeneration and Targeted Therapy

Advanced HMNs facilitate tissue regeneration and wound healing. Bismuth-nanosheet- and YAP signaling inhibitor (verteporfin, Vp)-loaded HMNs for wound healing reduced fibrosis, promoted scar-free tissue repair, and allowed for secondary skin element regeneration [99]. DNA HMNs loaded with hypoxia-derived extracellular vesicles significantly enhanced neuroprotection, reduced neuroinflammation, and promoted angiogenesis in ischemia–reperfusion injury models [70]. Furthermore, HMNs loaded with curcumin and β-cyclodextrin provided controlled drug release, improving curcumin solubility and anticancer efficacy [50].

Table 3 presents the functional properties of hydrogel microneedles (HMNs), covering key physicochemical characteristics. Swelling capacity varies widely (150–4000%), with PVA-, PVP-, and HA-based MNs showing high swelling, enabling enhanced drug loading and controlled release. Skin penetration depth ranges from 100 to 900 µm, influenced by MN composition and fabrication method. Mechanical strength varies from 0.1 to 1.5 N/needle, with crosslinked MNs exhibiting the highest robustness. Drug release kinetics range from rapid (HA, PEGDA) to sustained (GelMA, Dex-MA, PNIPAAm). Bioadhesiveness, permeation enhancement, and wearable sensor integration improve therapeutic efficiency. Smart MNs show photosensitivity, antibacterial properties, and glucose monitoring accuracy, highlighting their multifunctionality in drug delivery, diagnostics, and real-time biosensing applications.

## 5. Applications of HMNs in Drug Delivery and Diagnostics

HMN applications range from infectious disease treatment, chronic disease management, and cancer therapy to real-time diagnostic monitoring and biomarker analysis. This section comprehensively explores the primary purposes of HMNs in both drug delivery and diagnostics.

### 5.1. HMNs for Drug Delivery

#### 5.1.1. Treatment of Infectious Diseases

HMNs provide an effective transdermal approach for antibacterial, antiviral, and antiparasitic therapies, ensuring localized drug diffusion, sustained effects, and reduced systemic toxicity. In bacterial infections, SmartFilm-based HMNs loaded with rifampicin have demonstrated effective transdermal drug delivery, offering a promising alternative to traditional antibiotic administration [94]. Similarly, acyclovir-loaded polymer-blend HMNs enhance sustained antiviral effects, particularly for herpes simplex virus treatment [1].

For tuberculosis therapy, a microneedle-based cocktail of rifampicin, isoniazid, pyrazinamide, and ethambutol significantly enhances skin permeation, reducing the burden of oral drug administration [2]. Additionally, HMNs improve albendazole solubility and systemic absorption, making them highly effective against parasitic infections [53]. In bacterial skin infections, gelatin methacryloyl microneedles facilitate the controlled release of amoxicillin, ensuring targeted treatment against *Staphylococcus aureus* and *Escherichia coli* [65].

#### 5.1.2. Chronic Disease Management

HMNs also play a vital role in managing chronic diseases by enabling sustained drug release, improved systemic absorption, and enhanced patient compliance. For diabetes management, NIR-responsive black phosphorus microneedles deliver insulin in a glucose-sensitive manner, minimizing frequent injections [44]. Similarly, MIL-100(Fe)-loaded HMNs ensure sustained metformin release, optimizing long-term blood sugar control [54]. HMNs with phenylboronic acid-based crosslinking facilitate glucose-responsive insulin release, improving glycemic control without frequent injections [13].

**Figure 4:**
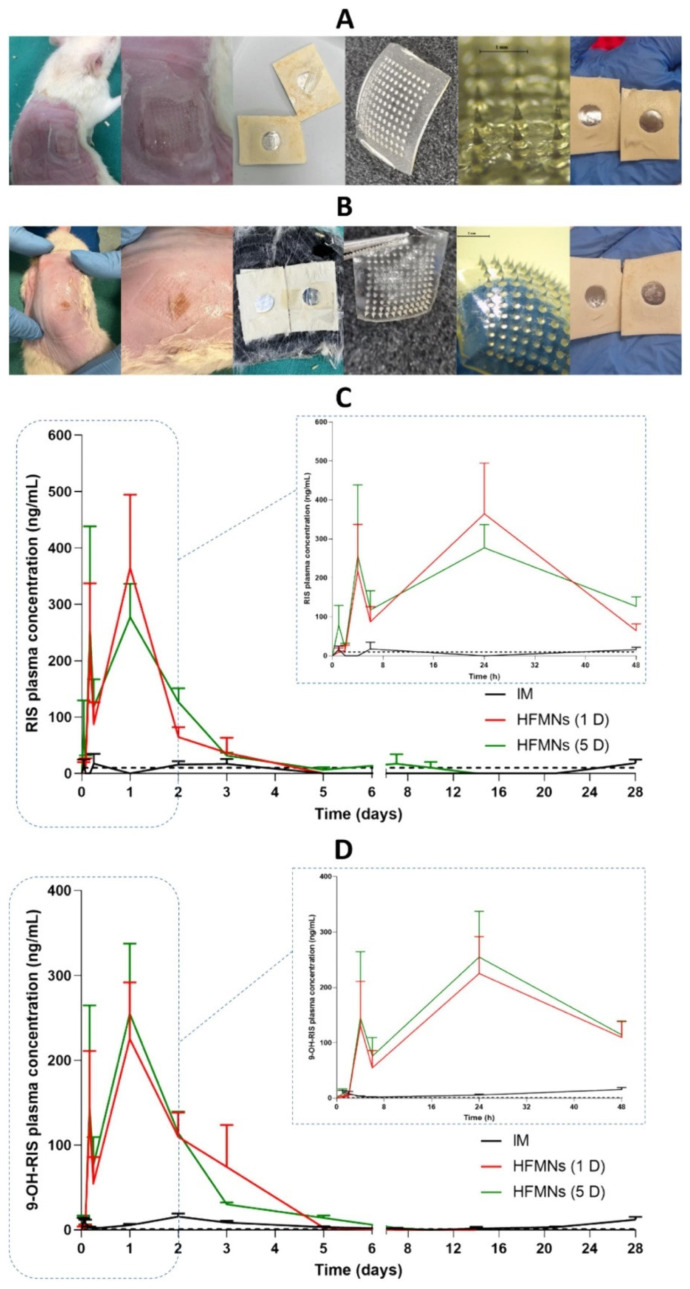
Images of the skin on the rats’ back, HFMAPs, and adhesive layer after the removal of patches at (**A**) 24 h and (**B**) 5 days and plasma profiles of (**C**) RIS and (**D**) 9-OH-RIS after a single-dose administration of 17 mg/kg (5 mg/rat) RIS from Risperdal Consta^®^ IM injection and HFMAPs (means + SDs, *n* = 9) at 1, 2, 4 and 6 h, *n* = 18 at the remaining time points. The black dashed line represents the LLOQ of the analytical method (10 ng/mL for RIS and 1 ng/mL for 9-OH-RIS) [57].

In the treatment of hypertension, HMNs enhance telmisartan solubility via beta-cyclodextrin complexes, ensuring higher drug permeation and bioavailability [49]. Similarly, HMNs with a direct compression tablet reservoir deliver sildenafil citrate, improving erectile dysfunction therapy by enhancing systemic absorption [56]. For schizophrenia management, HMNs allow for the prolonged systemic release of risperidone, eliminating the need for frequent oral doses and improving medication adherence [57]. Figure 4 depicts the in vivo study of hydrogel forming microneedle array patches (HFMAPs) to deliver RIS (risperidone).

#### 5.1.3. Wound Healing and Dermatological Applications

HMNs have proven highly effective in wound healing, anti-inflammatory therapy, and transdermal cosmetic applications. For instance, HMNs loaded with exosomes and tazarotene accelerate diabetic wound healing by stimulating cell migration, angiogenesis, and tissue repair [69]. In keloid prevention, dual-drug microneedles loaded with gallic acid and quercetin regulate fibroblast proliferation and oxidative stress, minimizing excessive scar formation [24]. Additionally, in anti-inflammatory therapy, HMNs deliver dexamethasone, diclofenac offering sustained localized relief for conditions such as psoriasis and actinic keratosis [6,63].

#### 5.1.4. Cancer Therapy and Precision Drug Targeting

HMNs facilitate localized cancer treatment, ensuring higher drug concentrations at tumor sites while minimizing systemic toxicity and side effects. In pre-cancerous skin lesions, HMNs improve the targeted deposition of 5-fluorouracil (5-FU), optimizing treatment for actinic keratosis and basal cell carcinoma [115]. Similarly, in colorectal cancer, lens-shaped microneedles enable deep light penetration, activating chemotherapeutics and improving photodynamic therapy outcomes [105]. Furthermore, for melanoma treatment, HMNs enhance TMPyP photodynamic therapy, ensuring targeted anticancer drug release with reduced systemic toxicity [4]. For liver cancer treatment, oncolytic Newcastle disease virus-loaded HMNs allow for localized viral release, improving cancer immunotherapy efficacy [116].

### 5.2. HMNs for Diagnostic and Biosensing Applications

#### 5.2.1. Real-Time Disease Monitoring and Biomarker Detection

HMNs provide a minimally invasive approach to real-time disease monitoring, offering continuous biomarker analysis and early disease detection. Peptide nucleic acid-integrated microneedles enable sequence-specific nucleic acid biomarker detection, providing precise diagnostics for cancer and genetic disorders [74]. For apoptosis and microbial biofilm studies, DNA aptamer-functionalized HMNs allow for the in situ fluorescent detection of cytochrome c, enabling the early detection of cell death and biofilm formation [36]. In diabetic monitoring, dopamine-functionalized HMNs track ketone levels, assisting in diabetic ketoacidosis prevention [26]. Additionally, in kidney function assessment, c-GelMA microneedles facilitate interstitial fluid extraction, enabling real-time urea concentration monitoring [66].

#### 5.2.2. Continuous Glucose Monitoring and Diabetes Management

**Figure 5:**
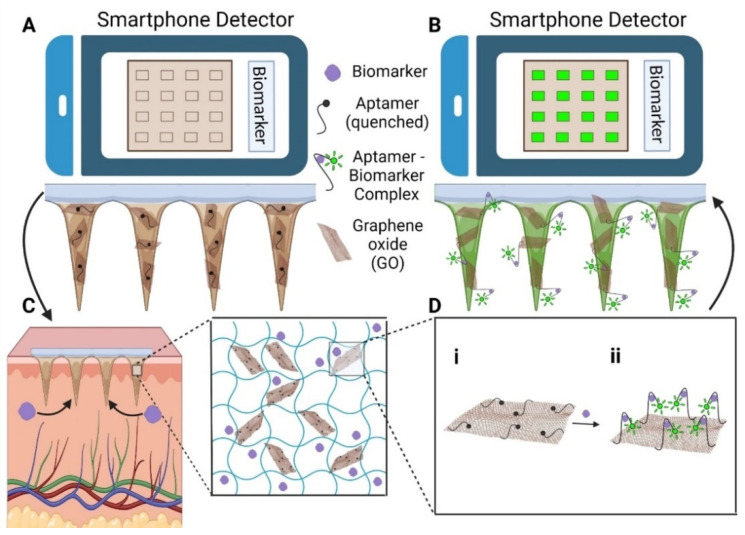
(**A**) To set the baseline fluorescence (F0), HMN-GO.NA patches are imaged with a smartphone before skin application. (**B**) After skin application, fluorescence increases to F, and the response is calculated as (F-F0)/F0. (**C**) The patches extract interstitial fluid (ISF) and biomarkers, with the inset showing the hydrogel network (MeHA, GO.NA, and biomarkers). (**D**) Schematic of the GO.NA sensing mechanism (**i**) before and (**ii**) after aptamer–biomarker complex formation [17].

HMNs have the ability to transform diabetes management by enabling continuous glucose monitoring and glucose-sensitive insulin delivery. In real-time glucose tracking, dextran-methacrylate microneedles integrated with bio-electroenzymatic sensors ensure the long-term monitoring of interstitial glucose levels [21]. Moreover, RF-coupled HMNs enable minimally invasive and wireless glucose detection, simplifying point-of-care diabetes management [117]. In biosensing applications, portable devices using HMN-integrated graphene oxide-nucleic acid (GO.NA) complexes enable the fluorescence-based detection of small molecules such as glucose, uric acid, insulin, and serotonin in interstitial fluid [17] (Figure 5).

#### 5.2.3. Early Disease Detection and Viral Diagnostics

HMNs enable non-invasive early disease detection, improving screening for viral infections and metabolic disorders. HMNs embedded with glypican-1 antibodies selectively bind tumor-derived exosomes, facilitating early colorectal cancer screening [96]. In ocular diagnostics, hyaluronic acid microneedles embedded in collagen cryogels offer sustained drug release and tear fluid analysis, providing a novel method for eye disease monitoring [51]. Furthermore, in neurological and physical assessments, Tb@ME-TPA-based PVA microneedles enable dopamine and lactic acid sensing, allowing for real-time biochemical monitoring via smartphone integration [29].

Table 4 highlights the diverse applications of HMNs in drug delivery and diagnostics, emphasizing targeted therapies, improved bioavailability, and enhanced patient compliance. Antibiotic, antiviral, and anti-inflammatory agents benefit from increased permeation and controlled release. Cancer therapy, psoriasis treatment, and metabolic disease management leverage MNs for localized and sustained drug administration. Diabetes, hypertension, and erectile dysfunction therapies demonstrate non-invasive transdermal delivery advantages. Neurodegenerative and hormonal therapies benefit from prolonged drug release, while gene delivery and vaccination enable immunotherapy applications. In diagnostics, real-time glucose, biomarker, and blood monitoring via wearable biosensors showcases MNs’ integration with personalized medicine. The table highlights MNs’ role in transdermal therapy, biosensing, and controlled drug delivery, addressing various clinical and healthcare challenges.

## 6. Testing Framework for HMNs

HMNs’ successful translation into clinical and commercial use depends on rigorous testing to ensure mechanical integrity, drug delivery efficiency, biosensing capabilities, and patient safety and compliance.

### 6.1. Physicochemical and Mechanical Characterization

The mechanical strength of HMNs is crucial to their effectiveness, as they must penetrate the skin without fracturing or losing efficacy. Fracture force assessments, penetration ability tests, and insertion efficiency studies using porcine skin, parafilm, and human cadaver skin models provide valuable insights into their robustness [1,19,43,52,66,94,103]. Optical coherence tomography (OCT) further enables the real-time imaging of microneedle insertion and structural integrity [104]. Since HMNs rely on fluid absorption for biomarker extraction and drug diffusion, their swelling kinetics and hydration behavior must be assessed under biological conditions [10,22,41,128]. Hydrophilicity studies confirm interstitial fluid (ISF) extraction efficiency, a critical factor for biosensing applications [33,129].

Physicochemical characterization techniques such as nuclear magnetic resonance (NMR), Fourier-transform infrared spectroscopy (FTIR), Raman mapping, X-ray diffraction (XRD), and differential scanning calorimetry (DSC) aid in evaluating crosslinking efficiency, molecular integrity, and phase transitions [19,20,21,49,130]. Stability testing under various temperature and humidity conditions determines long-term usability. For instance, studies have shown that HMNs stored at 20.0 ± 2.0 °C for three weeks at 43% relative humidity (RH) retained their mechanical strength, whereas those stored at 86% RH softened [91].

### 6.2. Drug Delivery Efficiency and Permeation Studies

To ensure effective transdermal drug delivery, HMNs are evaluated for drug release kinetics, permeability, and systemic bioavailability. Franz diffusion cells, in vitro and ex vivo skin models, and in vivo plasma concentration monitoring are commonly employed to assess permeation efficiency [2,10,35,60,125]. Pharmacokinetic studies in animal models provide essential data on systemic drug absorption and sustained-release profiles [15,39,49,95]. Sol-gel transition analysis and diffusion modeling aid in refining controlled drug release strategies to enhance therapeutic efficacy [86,106,131].

### 6.3. Disease-Specific Therapeutic Applications

In diabetes management, glucose-responsive HMNs undergo rigorous testing to confirm their ability to release insulin in response to fluctuating blood glucose levels [44,54,119,132]. These studies also evaluate oxidative stress protection and bioactivity retention under diabetic conditions to ensure the long-term therapeutic viability of HMNs [119]. Similarly, cancer therapy applications leverage near-infrared (NIR)-triggered drug release and photothermal therapy to achieve targeted chemotherapy, minimizing off-target effects [7,105]. Additionally, microneedle-based cancer vaccines are tested for antigen release kinetics, dendritic cell activation, and immunogenicity to evaluate their effectiveness in cancer immunotherapy [8,116].

### 6.4. Wound Healing and Infection Treatment

HMNs have also demonstrated potential for wound healing and infection treatment. Assessments include biofilm debridement tests, chronic wound healing efficacy studies, and tissue migration assays to evaluate their regenerative properties [25,69,98,118]. To ensure antimicrobial efficacy, HMNs are tested against common bacterial strains, including *Escherichia coli* and *Staphylococcus aureus* [91,109,110]. Additionally, nitric oxide (NO)-releasing HMNs are evaluated for their ability to provide infection control and wound protection through controlled antimicrobial agent release [31,109].

### 6.5. Optical Studies

For photo-switchable drug activation, HMNs undergo waveguide resorption studies and optical beam penetration depth tests to determine their efficiency in light-triggered drug release applications [105]. These studies are particularly relevant for therapies that rely on precise spatial and temporal control over drug activation.

Table 5 outlines the testing framework for evaluating HMNs, covering mechanical, pharmacokinetic, and biosensing assessments. Mechanical strength tests confirm that MNs withstand 0.5–1.5 N/needle, ensuring safe application. Swelling and absorption tests reveal swelling ratios up to 4000%, impacting drug release. Skin penetration studies confirm depths of 100–900 µm, with micropores closing within 24–48 h. Drug release and permeation studies show 3–50× transdermal enhancement, while histological analysis tracks MN morphology and tissue interaction. Pharmacokinetics indicate extended drug plasma half-life, improving efficacy. Biosensing validation ensures real-time glucose/biomarker monitoring with high sensitivity (>90%). Toxicity and in vivo studies confirm biocompatibility and patient compliance, demonstrating minimal pain and high acceptance in self-administration trials.

## 7. In Vivo and Clinal Trial Studies

### 7.1. In Vivo Studies (Animal Models)

Preclinical research on HMNs has demonstrated their effectiveness in various biomedical applications, including drug delivery, disease treatment, and diagnostics. In cancer therapy, HMNs have shown promising tumor suppression effects, often achieving synergistic therapeutic outcomes through controlled drug release [7,32,50]. For diabetes management, HMNs have successfully monitored glucose levels and facilitated insulin delivery, with real-time sensing capabilities comparable to commercial glucometers [15,16,40,117]. Similarly, in wound healing and regenerative medicine, HMNs have accelerated tissue repair, promoted angiogenesis, and exhibited strong antibacterial effects, making them valuable for chronic wound treatment [12,25,46,47,98,109].

Beyond these applications, HMNs have been explored for neurological and cardiovascular conditions, where they have demonstrated neuroprotection, angiogenesis promotion, and nerve regeneration in relevant animal models [70,84]. In dermatology, HMNs have contributed to pigmentation disorder treatments and hair regrowth, showcasing their potential in non-invasive skin therapies [37,79,92]. Furthermore, HMNs have played a role in immunotherapy and vaccine delivery by enhancing immune responses and providing an alternative to conventional injections [8,126]. Collectively, these preclinical findings validate the functional versatility of HMNs and support their continued development for clinical applications.

### 7.2. Clinical Studies in Humans

Although most HMNs remain in the preclinical phase, initial human trials indicate their safety, feasibility, and effectiveness. Studies have confirmed that HMNs can penetrate the skin without significant adverse effects, with skin barrier function recovering within hours post application [34,35]. Their self-application potential has also been demonstrated, showing high user success rates and no systemic infections or microbial penetration beyond the epidermis [91,100]. Furthermore, repeat applications have been found to cause no prolonged inflammatory responses, suggesting their suitability for routine use [112].

## 8. Advances and Achievements in HMN Technology

### 8.1. Enhancing Transdermal Drug Delivery and Bioavailability

HMNs have demonstrated significant improvements in drug permeation and bioavailability, offering a viable alternative to traditional oral and injectable therapies. Several studies have reported enhanced transdermal delivery efficiency, leading to higher drug deposition and therapeutic efficacy. For example, rifampicin-loaded HMNs achieved a four-fold increase in skin deposition, improving tuberculosis treatment [94]. Similarly, acyclovir-loaded MNs exhibited a 39-fold increase in drug permeation, ensuring sustained antiviral effects [1].

Other studies highlight successful applications in chronic disease management. Meanwhile, sildenafil citrate MNs bypassed first-pass metabolism, achieving 80% bioavailability, thereby enhancing erectile dysfunction treatment [52,56]. Additionally, telmisartan-loaded MNs demonstrated a 20-fold increase in solubility, optimizing pharmacokinetics for antihypertensive therapy [49].

Moreover, sustained and controlled drug release has been a major advantage of HMNs. Cyclodextrin-based risperidone MNs enabled systemic schizophrenia treatment with prolonged drug release for up to 10 days, reducing the frequency of dosing and improving patient adherence [57]. Similarly, double-network GelMA-ACNM MNs facilitated burst and extended drug release, making them suitable for combination therapies [67].

Smart and responsive HMNs are also transforming diabetes management and precision medicine. Photo-responsive MNs using black phosphorus enabled light-controlled insulin release, improving blood glucose regulation in diabetic models [44]. Glucose-sensitive MNs containing phenylboronic acid provided self-regulated insulin release, mimicking the function of pancreatic cells for more effective diabetes treatment [40]. Triple-responsive MNs responded to pH, temperature, and glucose levels, ensuring customized and precise drug release [90].

### 8.2. Biosensing and Smart Diagnostics: Revolutionizing Disease Monitoring

HMNs have also shown great promise in biosensing applications, enabling real-time non-invasive biomarker detection. One of the most impactful developments is the integration of biosensors with HMNs, allowing for continuous health monitoring. For instance, graphene oxide-integrated MNs detected multiple biomarkers, including glucose, insulin, serotonin, and uric acid, enabling multiplexed diagnostic capabilities in wearable health monitoring systems [17].

Innovative MN-based point-of-care diagnostics have been designed to improve disease detection speed and accuracy. Dual-layer MN patches with glucose oxidase provided colorimetric glucose detection, facilitating rapid and painless metabolic monitoring for diabetes [33]. Additionally, gold–copper nanosphere MNs enabled highly sensitive glucose detection from interstitial fluid, improving non-invasive glucose monitoring solutions [16].

Wireless and AI-integrated biosensing MNs have also emerged as a next-generation health monitoring system. Tb-functionalized MNs with smartphone-based fluorescence sensing enabled dopamine and lactic acid monitoring, expanding applications for neurological and metabolic disease tracking [29].

Another breakthrough in hydrogel MN diagnostics is their ability to rapidly extract and analyze interstitial fluid (ISF). Hexagram-shaped MNs with ultrasonic assistance enhanced ISF extraction, optimizing biomarker detection for psoriasis [111]. Osmosis-powered MNs facilitated fast ISF collection for real-time glucose and insulin analysis, improving accessibility to minimally invasive diagnostics [93].

### 8.3. Regenerative Medicine and Wound Healing Innovations

**Figure 6:**
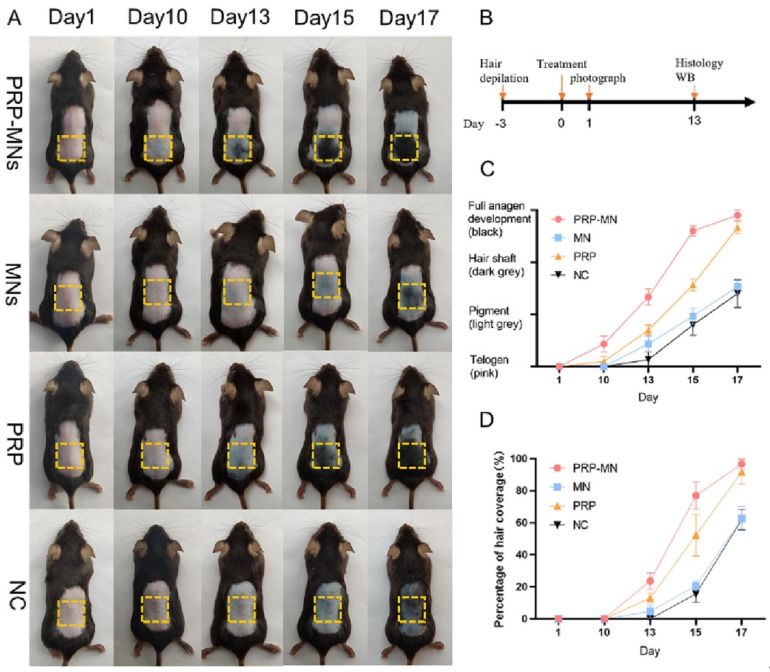
RP-MNs induce hair regrowth in vivo. (**A**) The treated dorsal skin was photographed at 1, 10, 13, 15, and 17 days after the administration of PRP-MNs. (**B**) Schematic representation of the animal experiments. (**C**) Hair phenotype transformed over time. (**D**) Percentage of hair coverage in the treated area. Adopted with permission [92].

HMNs can potentially influence wound healing, tissue regeneration, and immunotherapy applications by integrating biomimetic, exosome-loaded, and drug-releasing materials. One of the most exciting breakthroughs is exosome-loaded hydrogel MNs, which facilitate cellular regeneration and immune modulation [69]. PRP-loaded MNs promoted hair regrowth in alopecia patients by stimulating angiogenesis and dermal proliferation (Figure 6) [92]. Similarly, mesenchymal-stem-cell-derived exosome MNs significantly reduced neuroinflammation and promoted angiogenesis, aiding in ischemia–reperfusion injury recovery [70].

In chronic wound healing, biofilm disruption and antibacterial properties have been crucial. MgO@polydopamine-loaded MNs exhibited antioxidant, antibacterial, and pro-angiogenic effects, accelerating infected wound healing [73]. HMNs with nitric oxide-releasing properties effectively disrupted bacterial biofilms and promoted tissue repair, making them a powerful tool for wound management [109]. Bacterial-responsive MN dressings with doxycycline-loaded tips further enhanced chronic wound healing, ensuring targeted antimicrobial action [98].

Moreover, HMNs have improved vaccination efficacy. Methacrylated hyaluronic acid MNs combined with laponite nanocomposites facilitated sustained antigen release, enhancing immune response for vaccine applications [8]. Meanwhile, HMNs encapsulating oncolytic Newcastle disease virus selectively killed liver cancer cells, demonstrating potential for minimally invasive cancer immunotherapy [116].

### 8.4. Material Engineering and Fabrication Innovations

Significant advancements in microneedle fabrication techniques have led to improved mechanical performance, scalability, and patient compliance. High-precision 3D-printed MNs with porous structures enabled cost-effective and multifunctional drug delivery [89]. Infrared-processed MNs were developed for scalable and stable melanoma therapy, ensuring large-scale manufacturability [32].

Efforts to develop biodegradable and eco-friendly MNs have also advanced. Fish-scale-derived hydrogel MNs provided sustainable room-temperature crosslinking, ensuring environmentally friendly manufacturing while maintaining mechanical stability [130]. Additionally, calcium sulfate–gelatin composite MNs demonstrated high porosity and prolonged hypoglycemic effects, making them an ideal insulin delivery system [132].

Another key breakthrough is self-administration and patient-friendly MN technologies. HMNs that were successfully self-applied by volunteers demonstrated high feasibility for home-based drug delivery [100]. HMNs with sildenafil citrate reservoirs improved patient compliance for erectile dysfunction treatment, eliminating the need for oral medications [56,121].

## 9. Challenges Limiting Clinical and Commercial Adoption

Despite their transformative potential in drug delivery, diagnostics, and regenerative medicine, HMNs face several technical, regulatory, and clinical limitations that must be addressed for widespread adoption.

### 9.1. Challenges in Drug Release Precision and Control

One of the primary challenges of HMNs is achieving precise and consistent drug release, particularly for high-dose and stimuli-responsive formulations. Drug release is highly influenced by hydrogel composition, crosslinking density, and external conditions, leading to variability in encapsulation and controlled release kinetics [2,20,28].

Burst release remains a concern, particularly in high-dose formulations such as tuberculosis (TB) drugs and melanoma treatments (doxorubicin), where maintaining a steady therapeutic concentration is critical for efficacy [2,68]. Additionally, light-triggered insulin release in photo-responsive MNs requires further optimization to prevent unintended drug leakage [44].

Moreover, there is an inherent upper limit to the amount and kind of drugs that can be loaded into microneedles, posing a major constraint for formulations requiring high drug concentration. This limitation is particularly evident in hydrogel-based MNs, where drug encapsulation is restricted by swelling properties and material porosity. Recent advancements in polymeric porous microneedles, such as those made from biocompatible and photo-curable resin, offer a potential solution by enabling the loading of solid drug formulations in concentrated forms, as demonstrated with anesthetics and anti-inflammatory drugs like Lidocaine and Ibuprofen [135]. However, further studies are needed to validate the controlled release behavior of these porous microneedles in long-term clinical applications.

### 9.2. Mechanical Strength, Structural Integrity, and Skin Penetration Issues

HMNs must balance sufficient mechanical strength and swelling efficiency to ensure proper skin penetration without breakage. However, excessive swelling may lead to loss of mechanical integrity, limiting needle insertion efficiency and depth control. Certain swelling-based hydrogel MNs, such as pectin-enhanced MNs, may over-expand after application, which can negatively impact their biosensing and sampling accuracy [101]. Soft HMNs, including biodegradable and bacterial-responsive variants, are particularly vulnerable to deformation during application, requiring additional reinforcement strategies [22,79,109].

### 9.3. Stability and Scalability Challenges

HMNs are highly sensitive to moisture and environmental conditions, affecting storage stability, usability, and shelf life [35,91]. Crosslinking techniques such as gamma irradiation or template molding enhance performance and structural stability, but these processes increase production costs and may affect drug retention properties [58].

Additionally, MOF-integrated HMNs—which have shown promise in biomarker detection and smart drug release—face long-term stability challenges due to the degradation of the material over time [136]. Similarly, temperature-sensitive swelling MNs must be formulated carefully to avoid alterations in drug release profiles under varying environmental conditions [106].

From a manufacturing standpoint, advanced 3D printing and photopolymerization techniques enhance precision and drug delivery control but increase fabrication costs, making commercialization financially challenging [19,28,65]. Hybrid MNs that integrate biosensors, AI-based diagnostics, and nanomaterials (such as terbium-functionalized MNs for smartphone-linked diagnostics) are also expensive to manufacture, further hindering large-scale adoption [29].

### 9.4. Biosensing Accuracy and Clinical Validation Barriers

While HMNs show tremendous potential for biosensing applications, their sensor accuracy and stability over time remain key challenges. Real-time glucose- and pH-monitoring MNs require further calibration studies before widespread clinical adoption, particularly for wearable continuous monitoring systems [29,80,81,119].

Exosome-extracting MNs for cancer diagnostics need extensive validation studies to confirm their sensitivity and specificity across different patient populations [96]. Similarly, smartphone-integrated MN biosensing platforms require standardization for universal compatibility, ensuring consistent and accurate readings [29].

Additionally, multiplexed biosensing MNs—such as graphene oxide–nucleic acid probes for simultaneous glucose, insulin, serotonin, and uric acid detection—must undergo rigorous real-world usability testing to validate their long-term reliability [17].

### 9.5. Regulatory, Clinical Translation, and Commercialization Barriers

The transition of microneedle (MN)-based technologies from research to clinical and commercial use faces significant hurdles. These challenges include regulatory approval, large-scale manufacturing, cost-effectiveness, and market readiness.

#### 9.5.1. Regulatory Hurdles

Regulatory approval remains a critical barrier in translating microneedle-based technologies to clinical practice. MNs, particularly those delivering drugs or vaccines, must undergo rigorous evaluation by regulatory agencies to ensure safety, efficacy, and quality control. The regulatory process involves multiple key aspects.

Ensuring biocompatibility and stability: Materials used in MNs must demonstrate long-term stability and safety to minimize skin irritation and systemic toxicity. The selection of suitable materials influences the final regulatory approval process, as non-biocompatible materials may lead to adverse reactions [13,50,73].

Achieving Good Manufacturing Practice (GMP) compliance: Scaling up production while maintaining consistency and sterility remains challenging, particularly for 3D-printed and hydrogel-based MNs that require precise fabrication processes [19,89,90].

Establishing pharmacokinetics and biodistribution profiles: MNs must provide predictable and controlled drug release profiles to meet regulatory expectations for pharmaceutical or vaccine delivery systems. This aspect is crucial for ensuring therapeutic efficacy and safety [7,49,125].

Navigating classification uncertainties: MNs designed for drug or vaccine delivery often fall between medical devices and pharmaceutical products, leading to complex regulatory pathways. The classification affects how MN-based products are evaluated, which regulatory framework applies, and the level of clinical evidence required for market approval [8,91].

#### 9.5.2. Cost Analysis and Manufacturing Scalability

The production of MN-based systems must balance cost-effectiveness with scalability. Several challenges impact manufacturing efficiency and economic feasibility.

Material costs: Many MN formulations rely on hydrogels, biodegradable polymers, and metallic nanoparticles. While these materials provide biocompatibility and controlled release properties, their high cost can hinder large-scale production [50,73].

Standardization of production techniques: Variability in fabrication processes, such as batch-to-batch inconsistency in 3D printing and polymer crosslinking, affects the reliability and performance of MNs. This variability increases production costs and may lead to regulatory challenges in demonstrating product uniformity [20,89].

Scaling-up limitations: While MN manufacturing at the laboratory scale is well established, transitioning to commercial-scale production introduces difficulties in maintaining structural integrity, drug-loading uniformity, and reproducibility. These factors impact the efficiency of MN-based drug delivery systems, requiring innovative solutions for mass production [32,90].

Cold chain storage requirements: Some MN-based vaccines and protein-based therapeutics require strict storage conditions, increasing distribution complexity. Maintaining temperature-sensitive stability in MN formulations can significantly elevate logistics costs, creating barriers to widespread adoption in low-resource settings [137].

#### 9.5.3. Market Readiness and Commercial Adoption

Despite promising preclinical and clinical findings, several factors hinder MN technology’s market penetration and widespread adoption.

Patient adherence and acceptance: MNs offer pain-free drug delivery, which is a key advantage over conventional injections. However, patient compliance is influenced by multiple factors, including wear duration, ease of application, and perceived effectiveness. If patients find MN patches uncomfortable or difficult to use, adherence rates may decline [6,57].

Healthcare provider training: The successful implementation of MN-based technologies depends on clinician familiarity and training, especially for MNs integrated into diagnostic and monitoring devices. Adoption in clinical settings requires education on proper MN application, patient counseling, and device interpretation [80].

Competition with conventional methods: MN-based drug delivery competes with well-established methods, including oral tablets, injections, and transdermal patches. While MNs offer advantages such as minimally invasive administration and enhanced drug bioavailability, healthcare providers and pharmaceutical companies must demonstrate clear benefits over existing drug delivery technologies to achieve market differentiation and commercial success [38,50].

## 10. Future Directions in Hydrogel Microneedle Research

Despite current challenges, ongoing research is advancing HMNs for AI-assisted drug delivery, real-time health monitoring, and wearable diagnostics. The following key areas highlight the future advancements that will shape the next generation of HMN technologies.

### 10.1. Hybrid and Multifunctional Microneedle Systems

Future microneedle platforms will integrate drug delivery with biosensing to enable real-time diagnostics and on-demand therapy. These theranostic MNs will monitor biomarkers and administer drugs based on physiological changes, enhancing treatment precision and patient outcomes [5,13,21,26,77].

Stimuli-responsive MNs that release drugs in response to pH, enzymes, or glucose levels are being developed for personalized medicine and disease-specific treatment [4,13,14]. Additionally, mitochondria-targeting MNs are being explored for metabolic and neurodegenerative disorders, offering a novel approach to transdermal therapy [138].

Immunotherapy-based MNs represent another promising area, with designs incorporating vaccines or cancer immunotherapies to strengthen immune responses. These advancements could improve long-term protection against viral infections and enhance cancer treatments [126].

### 10.2. Integration of Nanotechnology for Enhanced Drug Delivery and Stability

Nanotechnology is expected to play a critical role in improving drug solubility, stability, and targeted delivery in HMNs. Future developments will focus on nanoparticle-enhanced MNs, utilizing materials such as gold, graphene oxide, and polymeric nanocomposites to improve drug bioavailability and precision targeting [7,12,24,47,49].

Additionally, cyclodextrin carriers and metal–organic frameworks (MOFs) are being investigated to enhance drug stability and controlled release, which could improve the efficiency of HMNs in chronic disease management [12,49,136].

### 10.3. Smart and Wearable Microneedle Patches for Real-Time Health Monitoring

The development of smart wearable MN patches will significantly impact real-time diagnostics and continuous patient monitoring. Future MNs will integrate flexible electronics and wireless connectivity, allowing patient data to be transmitted to cloud-based platforms for remote healthcare monitoring [15,17,26,102].

Self-adaptive stimuli-responsive MNs triggered by light, temperature, or electrical signals are also in development, providing personalized drug administration that adjusts to patient needs in real time [44,58].

A major innovation in this space is the creation of smartphone-compatible biosensing MNs for the point-of-care monitoring of diseases such as diabetes, cardiovascular conditions, and infections. AI-driven MN platforms may analyze real-time biomarker data and adjust drug release autonomously, enabling a highly personalized approach to disease management [26,29].

### 10.4. Optimization of Mechanical Performance and Material Properties

Ensuring the mechanical stability and durability of MNs remains a priority. Research is focused on reinforcing MNs with biocompatible polymers and nanomaterials, improving their penetration efficiency while maintaining flexibility [58,103,130,136].

Advances in double-network hydrogel structures and nanoengineered polymer reinforcements will further enhance mechanical resilience and controlled swelling properties, preventing premature failure during skin insertion and drug release [67]. Additionally, optimized HMN encapsulation strategies will improve stability by preventing premature swelling, thereby extending shelf life [107].

Sustainability is another focus, with increasing research on biodegradable MNs made from natural polymers such as alginate, chitosan, and cellulose, which enable scalable and cost-effective production while minimizing environmental impact [50,75,132].

### 10.5. Large-Scale Clinical Trials and Regulatory Pathways

For HMNs to reach mainstream healthcare, multi-center clinical trials are necessary to validate their long-term safety, efficacy, and patient adherence [16,35,55,57,100,112]. Specific applications, such as glucose-responsive insulin MNs and transdermal cancer therapies, will require rigorous human testing before clinical adoption [40,105].

Regulatory agencies, including the FDA and EMA, must establish clearer approval pathways for biosensing-integrated MNs to accelerate commercialization [5,124,127]. As combined MN systems (e.g., drug–biosensor hybrids) become more prevalent, regulators will need to refine classification and safety guidelines for these dual-function devices [17,80,119].

Additionally, gene-delivering MNs for CRISPR-based therapies and transdermal vaccinations require faster regulatory processes, especially as genetic and personalized medicine therapies continue to advance [82].

### 10.6. Personalized and Precision Medicine Approaches

HMNs have significant potential in personalized medicine, particularly through dual-layer and multi-phase drug release MNs, which allow for both immediate and sustained drug delivery based on biomarker fluctuations [67]. These MNs will dynamically respond to real-time patient data, optimizing treatments for chronic diseases, infections, and metabolic disorders [84].

Another promising direction is personalized immunotherapy, where exosome-loaded MNs are used for cancer treatment and tissue regeneration. By utilizing patient-derived exosomes, these MNs can enhance immune response specificity [115,134].

Additionally, long-acting MN patches could be developed for opioid addiction treatment, offering sustained drug release to support medication-assisted therapy, reduce withdrawal symptoms, and improve treatment adherence [87].

### 10.7. Development of Sustainable and Cost-Effective MN Fabrication Techniques

Scalability remains a key challenge in HMNs manufacturing. Future research will focus on high-throughput fabrication techniques, including microwave-assisted synthesis, photopolymerization, and 3D printing, ensuring cost-effective production with high precision and reproducibility [20,28,65,105].

New methods, such as infrared processing, are emerging as viable solutions for mass production, providing stable drug delivery platforms at lower costs [32]. Additionally, integrating biocompatible nanomaterials will help reduce cytotoxicity while enhancing drug release precision and targeted therapy [50,76].

The use of plant-derived polymers (e.g., alginate, cellulose, and pectin) in HMNs is another area of interest. These materials support the development of low-cost biodegradable formulations, making advanced MN-based therapies accessible to a broader patient population [75,132].

## 11. Conclusions

HMNs represent a paradigm shift in transdermal drug delivery and biosensing, combining biocompatibility, precision drug release, and non-invasive biomarker monitoring. Despite their immense potential, challenges in stability, manufacturing scalability, and regulatory approval hinder their clinical integration. Advances in hybrid biomaterials, smart-responsive drug release, and AI-powered biosensing platforms offer promising solutions to these limitations. Future research must focus on multi-functional MNs, personalized medicine, and large-scale clinical validation to accelerate commercialization. As HMNs technology evolves, its integration into real-time health monitoring, chronic disease management, and controlled immunotherapy will redefine patient care. Bridging material innovation with regulatory advancements is the key to unlocking the full potential of HMNs in next-generation biomedical applications.

## Figures and Tables

**Table 1:** Key materials used for HMNs.

Key Materials and Additives	Notable Features	Reference
Polyvinyl alcohol (PVA)	Excellent swelling, biocompatibility, mechanical strength	[32,33,41,42,43,52,53,54,55,56]
Polyvinylpyrrolidone (PVP)	Strong hydrogel formation, rapid dissolution properties	[42,49,52,57,58,59]
Poly(ethylene glycol) (PEG) (various MWs)	Enhances microneedle flexibility and permeability	[1,2,6,39,60,61]
Gantrez S-97 (Poly(methylvinylether-co-maleic acid))	Super-swelling polymer, good mechanical properties	[34,39,61,62,63]
Methacrylated hyaluronic acid (MeHA)	Crosslinked for strong gel matrix, biosensor compatibility	[8,16,36,46,64]
Gelatin methacryloyl	Biodegradable, tunable crosslinking, mechanical robustness	[19,65,66,67,68,69,70,71]
Chitosan (CS)	Biocompatibility, antimicrobial, wound healing properties	[38,54,55,72,73]
Alginate (sodium alginate)	Hydrophilic, bioadhesive, tunable gel strength	[74,75,76]
Polyacrylamide (PAM)	Crosslinking enhances durability	[77,78]
Dextran methacrylate (DexMA)	Strong crosslinking, bioadhesive	[7,21,79]
Dopamine	Conductivity, redox properties, bioadhesion	[15,26,30]
Graphene oxide (GO)	Conductive, improves transdermal penetration	[17,58,80]
Phenylboronic acid (PBA)	pH-sensitive, reversible binding	[13,40,81]
Carbopol	Swelling property, enhances release, glucose/pH-responsive	[61,81,82]
Silver Nanoparticles (AuNPs, AgNPs)	Strong antibacterial effect, enhanced wound healing, electrocatalyst	[15,16]
Poly(ethylene glycol) diacrylate (PEGDA)	Tunable crosslinking density for sustained release	[69,77,83]
Poly(N-isopropylacrylamide) (PNIPAAm)	Smart polymer, thermo-responsive swelling	[45,84]
Poloxamer-based Hydrogels (P407, P188, PLGA-PEG-PLGA)	Temperature-responsive, enhanced skin permeability	[82,85,86,87]
Hyaluronic acid (HA)	Strong water retention, bioavailability enhancement	[51,88]

**Table 2:** Fabrication techniques for HMNs.

Fabrication Method	Advantages	Limitations	Cost	Biocompatibility	Ref #
Photopolymerization (UV or Visible Light Crosslinking)	- Enables precise microneedle design with tunable mechanical properties. - Rapid and reproducible fabrication process. - Compatible with various hydrogel materials.	- Requires photoinitiators, which may cause cytotoxicity and skin irritation. - Mechanical strength and crosslinking efficiency depend on exposure conditions.	Moderate to high (requires photoinitiators and precision equipment)	Requires assessment of photoinitiator safety for biocompatibility	[7,19,69,71]
Enzyme-Mediated Crosslinking (HRP/H_2_O_2_, Transglutaminase, etc.)	- Biocompatible with mild reaction conditions, ideal for sensitive bioactive compounds. - Allows for tunable mechanical strength and degradation rates by adjusting enzyme concentration.	- High enzyme costs and stability challenges limit large-scale production.	High (requires cold storage and purification)	Excellent (highly biocompatible due to mild reaction conditions)	[4,31]
3D Printing (SLA, DLP, Extrusion-Based, Vat Photopolymerization)	- High resolution and customizable, enabling patient-specific designs. - Supports complex architectures with tunable mechanical and drug release properties.	- Limited material compatibility for hydrogel formulations. - Expensive equipment and technical expertise required. - Slow printing speed limits mass production.	High (requires expensive 3D printers, specialized resins, and post-processing)	Requires careful polymer selection to ensure biocompatibility	[19,44,65,89]
Micromolding (Solvent Casting, Freeze-Drying, Embossing, etc.)	- Cost-effective and simple fabrication process. - A well suited for mass production and industrial scalability. - Compatible with a wide range of hydrogels.	- Requires precise mold fabrication and material optimization.	Low (mold fabrication is a one-time cost; subsequent production is inexpensive)	Excellent (widely used in biomedical applications with established biocompatible materials)	[21,45,68,92]
Chemical Crosslinking (Glutaraldehyde, PEG, Citric Acid, Carbodiimide, etc.)	- Provides strong mechanical integrity with tunable degradation properties. - Ensures long-term stability and controlled drug release. - Scalable for batch production.	- Requires careful selection of crosslinking agents to balance mechanical strength and biocompatibility. - Residual crosslinkers must be thoroughly removed to ensure safety.	Moderate (varies by crosslinking agent; some are expensive)	Requires careful purification to remove residual crosslinkers for biocompatibility	[11,52,59,60]
Thermosensitive Hydrogel Formation (PNIPAAm, Poloxamer-Based Gels, etc.)	- Self-gelling at body temperature without external stimuli. - Injectable and adaptable for various drug release profiles. - Suitable for large-scale production.	- Requires optimization to balance gelation kinetics and mechanical performance.	Low to moderate (raw materials are relatively inexpensive)	Requires polymer selection to ensure optimal biocompatibility	[45,86,87]
Microwave-Assisted Crosslinking	- Significantly reduces reaction time (e.g., by 5 h. - Energy-efficient alternative to traditional crosslinking methods. - Reduces the need for harsh chemicals.	- Requires optimization to prevent material degradation and ensure uniform heating at larger scales.	Low to moderate (energy-efficient but requires specialized microwave equipment)	Requires careful control to prevent overheating	[20]
Freeze-Thaw Cycling (Physical Crosslinking)	- No chemical additives needed, reducing toxicity concerns. - Enhances mechanical strength and water absorption capacity. - Cost-effective due to minimal material requirements.	- Requires multiple freeze-thaw cycles, increasing processing time and affecting scalability. - Optimization needed to balance swelling behavior and mechanical performance.	Low (no need for chemical additives, making it highly cost-effective)	Excellent (highly biocompatible as no chemical crosslinkers are used)	[47]
Effervescent or Bubble-Generating Mechanisms (NaHCO_3_/Citric Acid, etc.)	- Enhances microneedle separation after skin penetration. - Self-dissolving mechanism improves drug delivery efficiency. - Cost-effective with simple fabrication using inexpensive raw materials.	- Requires formulation optimization to control gas release and prevent unwanted effects. - Industrial-scale production needs further optimization.	Low (inexpensive raw materials and simple fabrication process)	Good (safe materials, but gas formation may cause localized irritation)	[71]
Hybrid MNs (Rigid Outer Shell + Hydrogel Core)	- Combines high mechanical strength with hydrogel swelling for sustained drug release. - Allows for precise control over drug delivery profiles. - Provides enhanced structural integrity while maintaining biocompatibility.	- Complex fabrication process reduces scalability. - Requires optimization for efficient material integration.	High (requires multiple fabrication steps and specialized materials)	Excellent (well-controlled drug release with biocompatible hydrogel materials)	[28,48]
Conductive Hydrogel Integration (PEDOT:PSS, Graphene Oxide, Metallic Nanoparticles, etc.)	- Enables biosensing, real-time monitoring, and electrochemical drug release. - Enhances mechanical strength and electrical conductivity. - Potential for advanced biomedical applications.	- Complex fabrication process reduces batch efficiency. - Requires careful selection of conductive materials to ensure biocompatibility.	High (conductive polymers and nanoparticles are expensive)	Moderate (some conductive materials have cytotoxicity concerns)	[15,16,80]
Osmosis-Powered MNs (Maltose, Sorbitol, etc.)	- Enhances interstitial fluid (ISF) extraction for diagnostic applications. - Functions without external power sources. - Highly scalable through batch manufacturing.	- Requires optimization of swelling kinetics for precise fluid extraction.	Low (simple, affordable materials widely available)	Excellent (biocompatible non-toxic materials used)	[93]

**Table 3:** Functional properties of HMNs.

Functional Property	Key Observations and Technical Data	Reference
Swelling Capacity	Most HMNs exhibit swelling ratios between 150 and 4000%, allowing for increased drug loading and controlled release. PVA-, PVP-, and HA-based MNs show high swelling (>1000%), while PEG and Dex-MA MNs offer moderate swelling (300–800%).	[3,39,53,61,62,91,100,101]
Skin Penetration Depth	From 100 to 900 µm, depending on MN composition and geometry; 3D-printed MNs typically reach 300–500 µm, while dissolving MNs exhibit lower penetration (~250 µm) due to degradation.	[19,51,66,102,103,104]
Mechanical Strength (Force per Needle)	MN mechanical strength varies from 0.1 to 1.5 N/needle, with crosslinked GelMA, PVA, and PEGDA MNs exhibiting higher strengths (>0.5 N/needle), ensuring penetration without fracture.	[28,38,52,56,66,89,105,106]
Drug-Loading Capacity	Varies widely based on polymer type and MN structure: low-molecular-weight drugs (e.g., caffeine, lidocaine): 5–50 µg/mg polymer; high-molecular-weight drugs (e.g., proteins, mAbs): 0.1–10 mg/mg polymer.	[2,21,32,39,41,42,83,107]
Drug Release Kinetics	Fast-release MNs (HA, PEGDA, alginate) deliver 50–80% in first 2–6 h; sustained-release MNs (GelMA, Dex-MA, PNIPAAm) provide controlled release over 24 h to several days.	[6,7,13,42,45,49,54,86]
Bioadhesiveness and Skin Retention	MNs with carbomer, gelatin, and chitosan enhance skin adhesion, ensuring prolonged contact and stable drug delivery. Retention time ranges from 30 min to 24 h, depending on formulation.	[11,62,75,108]
Permeation Enhancement (Compared to Conventional Patches/Gels)	HMNs increase transdermal drug permeation by 3–50×, with caffeine, diclofenac, and valsartan showing >6-fold improvement.	[6,59,85,94,95]
Photosensitivity (for Light-Activated Systems)	Photodynamic therapy (PDT)-based MNs (TMPyP, PPIX, verteporfin) exhibit peak drug activation under 405–650 nm wavelengths, ensuring deep tissue penetration for melanoma and skin cancer treatment.	[4,14,105]
Glucose Monitoring Sensitivity	Detection range: 0.02–6 mM, with accuracy comparable to commercial glucometers. Conductive MNs (GO, PEDOT:PSS) enhance electrochemical response, enabling rapid glucose detection.	[15,21,33,58,78,80]
Antibacterial and Antioxidant Properties	MNs containing silver nanoparticles, nitric oxide, and graphene oxide demonstrated >99% bacterial reduction, along with enhanced wound healing.	[47,73,109,110]
Interstitial Fluid (ISF) Extraction Efficiency	Extraction volumes range from 5 to 15 µL in 1–5 min, with HA-based MNs showing fastest extraction.	[17,64,74,93,111]
Wearable and Biosensor Compatibility	HMNs successfully integrated with electrochemical, fluorescence, and colorimetric sensors, enabling real-time biomarker monitoring for glucose, pH, and dopamine.	[5,15,30,80,112,113,114]

**Table 4:** HMN applications in drug delivery and diagnostics.

Application Area	Primary Target (Drugs, Therapeutics, Biomarkers, etc.)	Notable Features	Reference
Antibiotic Delivery	Amoxicillin, Cefazolin, Vancomycin, Rifampicin	Targeting bacterial infections, increased permeation via MNs	[2,61,65,94,110]
Antiviral Delivery	Acyclovir	Improved skin penetration for viral infection treatments	[1]
Anti-Inflammatory Agents	Dexamethasone, Diclofenac, Ibuprofen Sodium	Localized, controlled release, reduced systemic effects	[6,39,63,107]
Wound Healing and Regenerative Medicine	Growth Factors, Platelet-Rich Plasma (PRP), Taurine, Exosomes	Targeted delivery, enhanced healing, anti-inflammatory effects	[25,69,73,88,92,118]
Cancer Therapy (Chemotherapy, PDT, Immunotherapy)	Doxorubicin, Tazarotene, Methotrexate, Oncolytic Viruses	Controlled release, targeting tumor microenvironments	[7,36,38,68,116]
Psoriasis Treatment	Methotrexate, Nicotinamide, Puerarin	Sustained release, local effect, minimizing systemic toxicity	[38,71]
Diabetes Management	Insulin, Metformin, Glucose-Responsive Hydrogels	Pain-free self-administration, glucose-responsive drug release	[13,40,44,45,54,81,119]
Hypertension Therapy	Valsartan, Telmisartan, Captopril	Transdermal delivery to avoid first-pass metabolism	[49,59,120]
Erectile Dysfunction Treatment	Sildenafil Citrate	Increased bioavailability, patient-friendly application	[42,52,56,121]
Tuberculosis Therapy	Rifampicin, Isoniazid, Pyrazinamide	MN-assisted transdermal therapy for improved compliance	[2]
Pain Management (Analgesics, Anesthesia)	Lidocaine, Caffeine	Rapid drug action, enhanced skin permeability	[10,122,123]
Ophthalmic Drug Delivery	Antibacterial Formulations	Ocular-specific MNs, sustained drug release	[51]
Photodynamic Therapy (PDT)	Photosensitizers (TMPyP, PPIX)	Light-activated therapy, enhanced transdermal absorption	[4,14,105]
Melanoma Treatment and Skin Disorders	Asiatic Acid, Alpha-MSH, Azelaic Acid	Targeted skin treatment, enhanced drug penetration	[32,37]
Metabolic Disease Management	Lithium, Ketone Monitoring	Non-invasive patient monitoring	[26,124]
Neurodegenerative Disease Therapy	Caffeine, Dopamine-Based MNs	Targeting Alzheimer’s and Parkinson’s via transdermal delivery	[10,29]
Hormonal Therapy	Estradiol, Melatonin	Sustained hormone release for long-term management	[125]
Anti-Scarring and Skin Regeneration	Gallic Acid, Quercetin, Berberine	Dual-drug release, tissue regeneration	[24,108]
Gene Delivery and Vaccination	Plasmid DNA, Lymphoma Vaccine, mRNA	Gene expression, immunotherapy applications	[118,126]
Continuous Glucose Monitoring	Enzyme-Based Glucose Biosensors	Non-invasive, real-time glucose monitoring	[16,21,30,33,78,80,81]
Blood Biomarker Monitoring	Glucose, Lactate, Dopamine, CRP, IL-1β, TNF-α	Continuous health tracking, wearable biosensors	[5,15,29,55,93,96,114]
Real-Time Electrochemical Biosensors	Graphene-Based Sensors	High-sensitivity biomarker detection	[80]
Drug Monitoring and Personalized Medicine	Real-Time Detection of Methotrexate, Theophylline, Isoniazid	Targeted drug release, improved adherence	[62,127]

**Table 5:** Testing framework for performance evaluation.

Testing and Evaluation	Key Observations and Technical Data	Reference
Mechanical Strength Testing	Compression force: 0.1–1.5 N/needle. Breakage resistance: most MNs withstand > 0.5 N for safe application. Insertion force range: 0.1–5 N.	[6,14,19,28,38,52,56,83,89,103,105,106]
Swelling and Absorption Tests	Swelling ratios: 150–4000%. Hydrophilic polymers (PVA, PVP, HA, Dex-MA) show higher swelling. Time to full swelling: 5–30 min.	[3,33,39,53,61,62,100,101]
Skin Penetration and Insertion Efficiency	Depth: 100–900 µm. Efficiency: confirmed via parafilm, porcine skin, and human skin models. Micropore closure rate: 24–48 h post application.	[19,51,66,102,103,104]
Drug Release and Permeation Studies	Fast release: HA, PEGDA, alginate MNs (50–80% in first 2–6 h). Sustained release: GelMA, Dex-MA, PNIPAAm (24 h–weeks). Drug permeation enhancement: 3–50× vs. control.	[6,7,13,42,45,49,54,86]
Transdermal Drug Delivery Enhancement	HMNs increase transdermal permeation 3–50×. Drugs like caffeine, diclofenac, and valsartan show six-fold+ improvement. Permeation rates: 20–90% of drug over 24–48 h.	[6,59,85,94,95]
Histological and Microscopic Analysis	Needle morphology: SEM, AFM, OCT imaging. Micropore closure: tracked using OCT and TEWL analysis. Histology staining: Hematoxylin and Eosin (H&E), Masson’s Trichrome.	[10,34,66,100,103,104,111]
Pharmacokinetics and Pharmacodynamics	Peak plasma concentration monitoring for sustained drug release evaluation. Area under the curve increase: 2–10× for MNs vs. oral/intravenous administration. Plasma half-life: extended 1.5–6× with MNs.	[39,57,60,86]
Biomarker and ISF Extraction Studies	ISF extraction rates: 5–15 µL in 1–5 min. Common for glucose, lactate, urea, dopamine, cytokine monitoring. Recovery efficiency: 70–98% vs. blood sampling.	[5,15,17,64,74,93,111]
Biosensing and Diagnostic Validation	HMNs successfully integrated with sensors (fluorescence, electrochemical, colorimetric) for real-time biomarker tracking. Glucose detection range: 0.02–6 mM, accuracy > 90%.	[15,21,58,78,80,112,114]
Electrochemical and Optical Sensing	Sensitivity: detection limit 0.02–6 mM for glucose and other analytes, comparable to commercial glucometers. Response time: 5–10 min for in situ detection.	[15,21,33,58,78,80]
Antibacterial and Antioxidant Testing	MNs loaded with silver nanoparticles, nitric oxide, and graphene oxide achieved >99% bacterial reduction in wound healing studies. Biofilm eradication efficiency: 80–98%.	[47,73,109,110]
Light-Responsive and Photodynamic Therapy (PDT) Studies	Wavelength ranges: 405–650 nm. Used for melanoma, PDT treatments, and controlled drug release systems. Photothermal effect efficiency: 2–4× higher vs. standard light therapy.	[4,14,105]
Toxicity and Biocompatibility Studies	Cell viability: >70% in keratinocytes, fibroblasts, and endothelial cells. Evaluated through hemolysis, MTT assays, and in vivo toxicity studies. No significant inflammatory response detected in in vivo studies.	[21,38,62,98,126]
In Vivo Animal Models for Disease Treatment, Ex Vivo Model	Used in rats, mice, and porcine models for wound healing, diabetes, cancer therapy, and neurological disorders. Therapeutic efficacy vs. standard treatment: 1.5–5× improved.	[39,57,60,70,86,133,134]
Patient and User Compliance Testing	Self-administration trials confirm minimal pain, high acceptance in volunteers, and enhanced compliance vs. injections. Pain score reduction: 3–7 points vs. hypodermic needles. Application time: 5–20 s.	[34,100,104,111]

## Data Availability

No new data were created or analyzed in this study.
